# Reciprocal Pathways Linking Harsh Parenting and Conduct Problems in Early Childhood: The Mediating Role of Emotional Dysregulation

**DOI:** 10.1007/s10802-026-01473-8

**Published:** 2026-07-04

**Authors:** Ricardo Mellado

**Affiliations:** Social Research Institute, University College London, 55-59 Gordon Square WC1H 0NU, London, United Kingdom

**Keywords:** Harsh parenting, Emotional dysregulation, Conduct problems, Externalising behaviour, Longitudinal mediation, Random-intercept cross-lagged panel model

## Abstract

Externalising behaviour—such as aggression, impulsivity, and defiance—is a persistent form of childhood maladjustment, yet the mechanisms linking early risk factors to later outcomes remain debated. Research has focused on harsh parenting practices and children’s emotion regulation difficulties as key processes shaping conduct problems across early childhood. However, while prior research has predominantly examined parent-driven pathways, transactional models suggest that parent and child influences may be bidirectional. Using data from the UK Millennium Cohort Study (*N* = 16,328; 51% male), this study examined reciprocal longitudinal associations between harsh parenting, emotional dysregulation, and conduct problems across ages 3, 5, and 7. A random-intercept cross-lagged panel model (RI-CLPM) was used to test whether emotional dysregulation mediates associations between harsh parenting and conduct problems in both directions. Results provided evidence for reciprocal mediation processes: harsh parenting was associated with subsequent increases in emotional dysregulation and later conduct problems, and early conduct problems were associated with later emotional dysregulation and harsher parenting practices. Both indirect effects were statistically significant. The child-driven pathway (β = 0.055) was larger than the parent-driven pathway (β = 0.013), although both effects were modest in magnitude. Findings were robust to sensitivity analyses addressing measurement overlap and unmeasured time-varying confounding. These results extend prior research by demonstrating that emotional dysregulation mediates bidirectional parent-child processes, with asymmetry favouring child-driven effects. Findings are consistent with transactional models of parent–child dynamics and suggest that interventions targeting both harsh parenting and children’s emotion regulation difficulties may help disrupt escalating cycles of risk in early childhood.

**Supplementary Information:**

The online version contains supplementary material available at 10.1007/s10802-026-01473-8.

## Introduction

Harsh parenting practices, such as shouting, smacking, or frequent verbal reprimands, have been consistently associated with elevated levels of children’s conduct-related externalising problems, including aggression and oppositional behaviour (Deater-Deckard et al., 2012; Gershoff et al., 2010; Lansford et al., 2011; Wiggers & Paas, 2022). These coercive parent-child interactions are thought to promote stable patterns of conduct problems by heightening children’s emotional arousal, limiting opportunities to learn constructive emotion management strategies, and modelling coercive responses to conflict (Morris et al., 2007; Patterson, 2002). Over time, such patterns may become increasingly consolidated and generalise beyond the home to school and peer contexts. At the same time, transactional models propose that children’s early conduct problems may evoke harsher parental responses, creating reciprocal cycles of influence across development (Lansford et al., 2011; Sameroff, 2009; Sulik et al., 2015). However, the developmental mechanisms mediating these bidirectional associations—and the extent to which parent-driven and child-driven processes operate through similar pathways—remain incompletely understood.

One process that may help explain the bidirectional associations between harsh parenting and children’s conduct problems is emotion regulation—the set of processes through which individuals monitor and modulate emotional arousal in ways that support adaptive, goal-directed behaviour (Eisenberg et al. 2010b; Shaw et al. 2014). When these processes are compromised, children may experience emotion dysregulation, characterised by difficulties modulating emotional arousal - such as rapid shifts in affect, low frustration tolerance, heightened emotional reactivity, and difficulty recovering from distress (Shaw et al., 2014; Nigg, 2017). Such difficulties can interfere with children’s capacity to respond flexibly to environmental demands and may increase vulnerability to maladaptive behavioural responses in challenging situations.

A substantial body of research suggests that parenting practices play a central role in shaping children’s emerging emotion regulation capacities (Morris et al., 2007). In particular, harsh or punitive parenting has been consistently associated with heightened emotional arousal and poorer regulatory functioning in children (Wang et al., 2023; Wang & Qi, 2017). Such practices may limit opportunities for children to learn constructive emotion management strategies within parent–child interactions. Repeated exposure to parental anger and punitive discipline can increase emotional overarousal and undermine children’s ability to recover from distress, while overly controlling responses may encourage rigid suppression of negative emotions rather than flexible regulation (Chang et al., 2003; Coplan et al., 2002; Sroufe, 1997). At the same time, children’s difficulties in regulating emotion may exacerbate parent–child conflict and elicit harsher parental responses over time (Colman et al., 2006; Grolnick et al., 1999; Scaramella & Leve, 2004). When children exhibit frequent emotional outbursts, low frustration tolerance, or difficulty calming down, parents may become increasingly reactive or resort to more coercive disciplinary strategies, creating the potential for reciprocal and escalating cycles (Patterson, 2002; Sameroff, 2009).

In turn, difficulties in emotion regulation have been shown to predict later externalising problems in children, particularly conduct problems (Eisenberg et al., 2010). Although closely related, emotion dysregulation and conduct problems are conceptually distinct constructs. Emotion dysregulation refers to difficulties in the processes through which emotional arousal is monitored and modulated—for example, problems calming down when frustrated, rapid escalation of affect, or limited recovery from distress (Shaw et al., 2014). Conduct problems, by contrast, refer to observable behaviours that violate age-appropriate social norms, including physical aggression, defiance, and destructive behaviour (Goodman, 1997). The two constructs therefore operate at different levels of explanation: emotion dysregulation reflects an underlying regulatory process, whereas conduct problems represent behavioural manifestations that may, among other factors, emerge when regulation is compromised. When children struggle to modulate intense negative emotions, they are more likely to respond to frustration or interpersonal conflict with aggression or defiance, increasing vulnerability to conduct problems over time (Hill et al., 2006). Conversely, persistent engagement in conduct-related behaviour may further undermine the development of regulatory capacities, as repeated conflictual interactions heighten emotional arousal and reinforce reactive response patterns (Eisenberg et al., 2010).

## Emotion Dysregulation as a Developmental Mechanism Linking Parenting and Conduct-related Externalising Problems

To address questions of temporal ordering and developmental mechanisms linking parenting practices, self-regulation, and externalising behaviour, prior research has frequently relied on longitudinal mediation models (Cole & Maxwell, 2003) to examine whether children’s self-regulatory capacities account for associations between early parenting and later externalising outcomes. This research agenda has primarily focused on cognitive and behavioural dimensions of self-regulation, such as effortful control, attentional control, and executive functioning. For example, Eisenberg et al. (2005) found that children’s effortful control mediated the association between early maternal warmth and later conduct-related externalising problems. Similarly, Belsky et al. (2007) reported that maternal sensitivity predicted later attentional control, which in turn was associated with lower levels of externalising-related traits, including anger, frustration, and aggressive behaviour, providing support for a predominantly parent-driven pathway from caregiving to child regulation.

Other studies have pointed to more complex developmental cascades involving parenting, self-regulation, and externalising behaviour. Using multiple waves of early childhood data, Sulik et al. (2015) found that executive functioning mediated associations between observed early caregiving behaviours and later conduct problems (indexed using the SDQ Conduct Problems scale) but also identified evidence for alternative pathways in which early conduct problems predicted subsequent self-regulatory capacities. Together, these findings indicate that parenting, self-regulation, and externalising behaviour may be linked through both parent-driven and child-driven processes across development.

Notably, however, far less attention has been paid to emotional dimensions of self-regulation, including the modulation of emotional arousal and the capacity to down-regulate negative affect, and to harsher forms of parenting that may place direct demands on children’s emotion regulation capacities. Much of the existing longitudinal mediation and cascade literature has focused on relatively supportive or sensitive caregiving behaviours, such as warmth, sensitivity, and cognitive scaffolding, and on children’s cognitive and behavioural regulatory skills, including effortful control and executive functioning (e.g., Eisenberg et al., 2005; Belsky et al., 2007; Sulik et al., 2015). Although developmental theory has long implicated emotion dysregulation as a key mechanism linking harsh or coercive parenting practices to children’s conduct-related externalising behaviour (Belsky et al., 2007; Morris et al., 2007), this process has rarely been examined empirically as a mediator within longitudinal developmental cascade models. With the exception of early work by Chang et al. (2003), prior research has largely prioritised cognitive regulatory capacities over children’s emotional reactivity and recovery from distress. As a result, emotion dysregulation remains largely untested as a mediating mechanism through which harsh parenting and conduct-related externalising behaviour become dynamically linked across childhood — despite strong theoretical grounds for expecting it to play such a role (Morris et al., 2007).

### This Study

The present study examines the longitudinal interplay between harsh parenting, emotional dysregulation, and conduct-related externalising behaviour across early to middle childhood (ages 3 to 7), testing within-person developmental pathways using random-intercept cross-lagged panel models. The developmental window of ages 3 to 7 was selected because it spans a critical period for the consolidation of emotion regulation capacities (Eisenberg et al., 2010; Sroufe, 1997) and because repeated measures of all three constructs—harsh parenting, emotional dysregulation, and conduct problems—were available at each wave. The CSBQ emotional dysregulation items were administered at ages 3, 5, and 7 but not at later MCS sweeps, constraining the longitudinal window to this period. Importantly, this developmental stage is theoretically significant: emotion regulation skills are undergoing rapid development during the preschool and early school years, parenting practices remain a primary socialisation influence, and early conduct problems are emerging but not yet consolidated — making this a period when reciprocal processes between these domains are most likely to be detectable and most amenable to intervention. The SDQ conduct problems subscale additionally uses age-appropriate wording at age 3 within the MCS, supporting its developmental suitability across this window.

The present study extends prior cascade research by examining emotional dysregulation — rather than cognitive self-regulation — as a mediator, and harsh parenting — rather than parental warmth or sensitivity — as the parenting dimension, testing whether these constructs are linked through regulatory capacities more directly implicated in emotionally charged and coercive parent-child exchanges. Specifically, we address the following research questions:

- **RQ1**: Does early harsh parenting predict later conduct-related externalising behaviour indirectly via children’s emotional dysregulation?
- **RQ2**: Does early conduct-related externalising behaviour predict later harsh parenting indirectly via children’s emotional dysregulation?

By examining both pathways, the study evaluates whether emotional dysregulation functions as a mediator in parent-driven, child-driven, or reciprocal developmental processes across childhood. Although prior research has suggested that self-regulatory processes may mediate associations between parenting and conduct problems, the present study remains agnostic regarding the direction and significance of longitudinal associations, given the distinct constructs examined (emotional dysregulation in the context of harsh parenting, rather than cognitive regulation in supportive caregiving contexts).

In addition to its conceptual contribution, the present study advances methodologically beyond prior work in three ways. First, the multi-wave design allows a full longitudinal test of mediation in which all constructs are adjusted for their prior levels (Cole & Maxwell, 2003), unlike two-wave designs that cannot isolate indirect pathways across successive time intervals (e.g., Chang et al., 2003). Second, the adoption of a random-intercept cross-lagged panel modelling (RI-CLPM) framework separates stable between-family differences from within-child fluctuations, providing a more appropriate test of developmental theories positing reciprocal within-family processes (Hamaker et al., 2015). Third, the use of a large, nationally representative birth cohort strengthens generalisability beyond high-risk or regionally specific samples.

## Method

### Data

Participants were drawn from the Millennium Cohort Study (MCS; Connelly & Platt, 2014), a UK-based longitudinal birth cohort study following children born between September 2000 and January 2002. The baseline sample comprised 19,244 families, including 19,517 children, from across England, Wales, Scotland, and Northern Ireland. Eligibility was based on residency in the UK at 9 months of age and receipt of Child Benefit, a near-universal welfare provision at the time. The MCS employed a stratified, clustered sampling design to ensure adequate representation of socioeconomically disadvantaged and ethnic minority groups (Hansen, 2014; Plewis et al., 2007).

For the present study, data were drawn from Waves 2 (age 3), 3 (age 5), and 4 (age 7) of the Millennium Cohort Study, with achieved samples of 15,808, 15,460, and 14,043 cohort members at each wave, respectively. The analytic sample comprised 16,328 children with valid data on at least one study variable across waves 2–4. At baseline (age 3), the sample was evenly distributed by gender (50.9% male), predominantly White (83.2%), and had a mean household income of 1.41 (SD = 0.28) on the OECD equivalised income scale, where values are standardised relative to household composition and higher scores indicate greater equivalised household income.

To examine potential attrition bias, participants present at wave 2 who were retained across all three waves (*n* = 12,180) were compared with those present at wave 2 with incomplete data at subsequent waves (*n* = 3,399) on baseline demographic characteristics and study variables. Participants with incomplete data were slightly more likely to be male (52.7% vs. 50.6%, χ² = 4.6046, *p* = .03) and from non-White ethnic backgrounds (23.5% vs. 14.9%, χ² = 138.37, *p* < .001). They also had marginally lower household income (M = 1.33, SD = 0.32 vs. M = 1.42, SD = 0.28, *p* < .001) and slightly higher baseline conduct problems (M = 2.98, SD = 2.14 vs. M = 2.79, SD = 2.05, *p* < .001) and emotional dysregulation (M = 1.92, SD = 0.46 vs. M = 1.87, SD = 0.45, *p* < .01). Differences in harsh parenting were small despite statistical significance (M = 9.09, SD = 2.51 vs. M = 9.39, SD = 2.37, *p* < .001). Overall, effect sizes were small (Cohen’s d < 0.15), consistent with typical attrition patterns in large longitudinal cohort studies. Missing data were handled using full information maximum likelihood (FIML), which incorporates all available observations and produces unbiased estimates under a missing-at-random assumption (Enders, 2022).

### Measures

Children’s conduct-related externalising behaviour was measured using the Conduct Problems subscale of the Strengths and Difficulties Questionnaire (SDQ). This subscale was administered in waves 2, 3 and 4 of the Millennium Cohort Study (MCS) and consists of five parent-reported items: “Often has temper tantrums or hot tempers,” “Generally obedient, usually does what adults request” (reverse-coded), “Often fights with other children or bullies them,” “Often lies or cheats,” and “Steals from home, school, or elsewhere.” Items are rated on a three-point scale (“Not True,” “Somewhat True,” “Certainly True”) and summed such that higher scores indicate greater conduct problems. To ensure developmental appropriateness, the SDQ age-3 version uses age-specific item wording, replacing “often lies or cheats” with “argumentative with adults” and “steals from home, school, or elsewhere” with “can be spiteful”. Prior research has established the SDQ’s strong psychometric properties, with the conduct subscale showing robust factor loadings (Bridger Staatz et al., 2024). Recent work with the MCS has further demonstrated longitudinal and gender measurement invariance for this subscale across early to middle childhood (Murray et al., 2024). Internal consistency in the MCS has been shown to be acceptable, with omega coefficients of 0.74 at age 3, 0.75 at age 5, and 0.79 at age 7 (Speyer et al., 2022).

Harsh parenting was assessed using three items from the Conflict Tactics Scale (CTS; Straus, 2017), measuring frequent verbal and physical disciplinary responses, such as shouting, smacking, and telling off when the child misbehaved. In the Millennium Cohort Study, primary caregivers (primarily mothers, approximately 98%) reported how often they used each tactic on a five-point Likert scale (“never,” “rarely,” “once a month,” “at least once a week,” “daily”). Responses were summed to create a continuous harsh parenting score, with higher values reflecting more frequent use of these coercive disciplinary practices. Prior research using the MCS has demonstrated longitudinal measurement invariance for this measure across ages 3, 5, and 7, as well as acceptable internal consistency, with omega coefficients ranging from 0.75 to 0.76 across waves (Speyer et al., 2022), supporting its reliability and longitudinal comparability across early childhood. These three items were selected from the broader set of disciplinary items available in the MCS because they capture coercive and punitive parenting responses — shouting, smacking, and verbal reprimands — consistent with the conceptual definition of harsh parenting used in prior research (Gershoff et al., 2010). The remaining items assessed non-punitive strategies (e.g., ignoring, removing privileges, time-out) that do not align with the harsh parenting construct. This operationalisation is consistent with prior MCS-based research examining harsh disciplinary practices in early childhood (Speyer et al., 2022; Rajyaguru et al., 2019).

Emotional dysregulation was assessed using five parent-reported items from the Child Social Behaviour Questionnaire (CSBQ) (Hartman et al., 2006), administered in waves 2, 3, and 4 of the Millennium Cohort Study. Parents rated how well each item described their child’s typical behaviour on a three-point scale (“not true,” “somewhat true,” “certainly true”), and responses were summed such that higher scores indicated greater emotional dysregulation. Items captured key features of dysregulation in young children, including mood instability (“shows mood swings”), heightened emotional reactivity (“gets over-excited”), low frustration tolerance (“easily frustrated”), impulsivity (“acts impulsively”), and difficulty recovering from distress (“gets over being upset quickly”). The latter item was reverse-coded, as it reflects regulatory capacity rather than dysregulation. Prior work has supported the reliability and validity of this subscale in population-based samples (Hartman et al., 2006). Within the MCS, the emotional dysregulation scale has demonstrated acceptable internal consistency, with omega coefficients of 0.78 at age 3, 0.77 at age 5, and 0.79 at age 7, as well as longitudinal and gender measurement invariance (Murray et al., 2024). Descriptive statistics for emotional dysregulation, harsh parenting, and SDQ conduct problems are presented in Table 1.

**Table 1:** Descriptive statistics for harsh parenting, emotional dysregulation, and conduct problems across three waves

Wave	*N*	Mean	SD	Min	Max	Skew	Kurtosis
Harsh Parenting							
Wave 2 (Age 3)	12,985	9.33	2.40	3	15	-0.11	-0.49
Wave 3 (Age 5)	14,131	8.35	1.97	3	15	0.09	-0.19
Wave 4 (Age 7)	13,001	8.10	1.91	3	15	0.13	-0.22
Emotional Dysregulation							
Wave 2 (Age 3)	14,431	1.88	0.45	1	3	0.06	-0.64
Wave 3 (Age 5)	14,522	1.73	0.46	1	3	0.38	-0.50
Wave 4 (Age 7)	13,280	1.73	0.48	1	3	0.39	-0.54
Conduct Problems							
Wave 2 (Age 3)	14,380	2.82	2.07	0	10	0.73	0.26
Wave 3 (Age 5)	14,501	1.52	1.52	0	10	1.18	1.61
Wave 4 (Age 7)	13,269	1.39	1.55	0	10	1.38	2.29

### Statistical Analysis

The hypothesised longitudinal mediation model was evaluated using structural equation modelling (SEM) in Mplus 8.11 (Muthén & Muthén, 2017). The analytic strategy followed the framework proposed by Cole and Maxwell (2003) for testing mediation in longitudinal designs, which emphasises temporal ordering across multiple waves—such that predictors precede mediators and mediators precede outcomes—while adjusting for prior levels of all constructs.

**Fig. 1:**
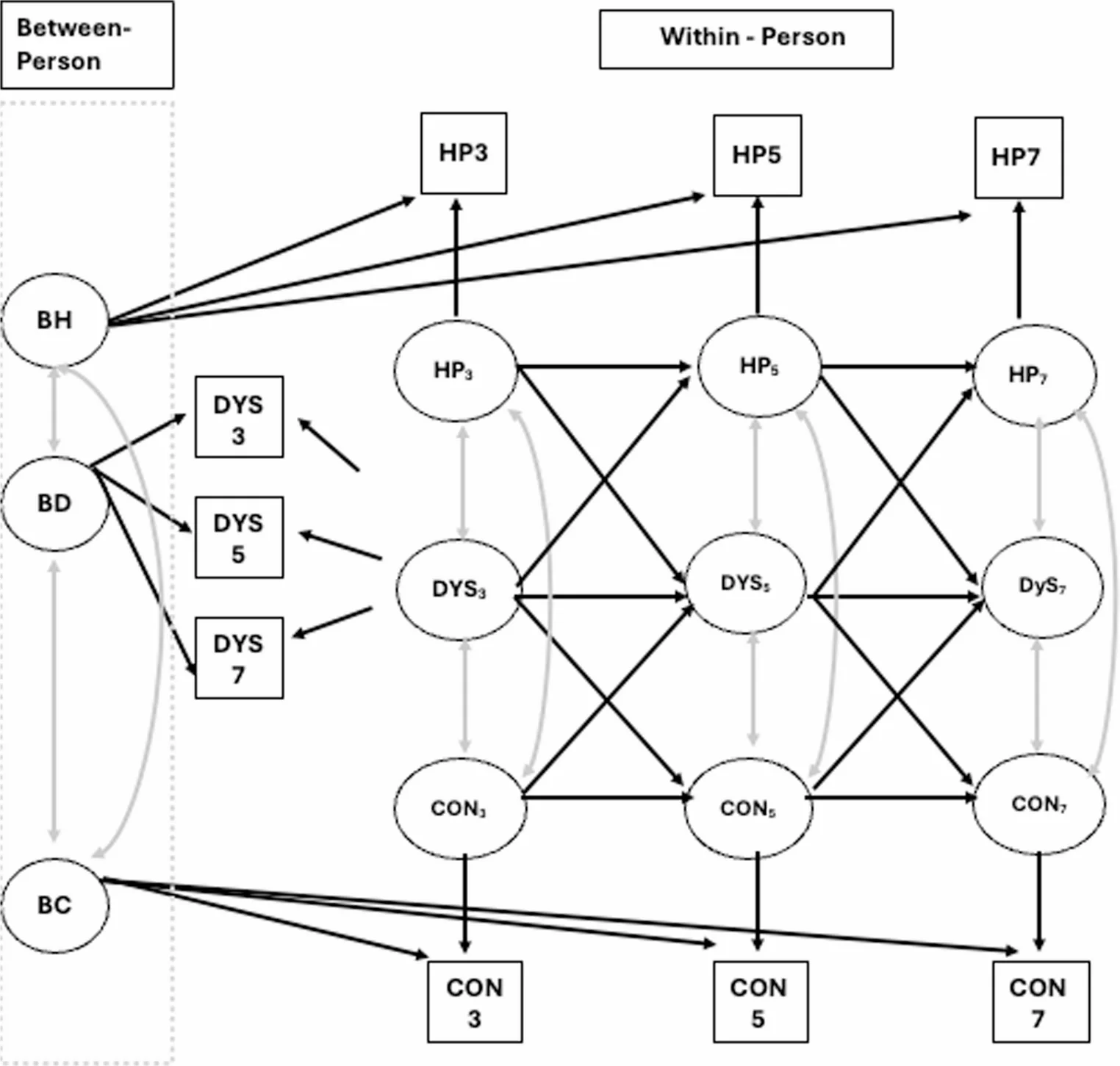
Conceptual diagram of the three-wave random-intercept cross-lagged panel model (RI-CLPM) illustrating longitudinal associations between harsh parenting (HP), emotional dysregulation (DYS), and conduct problems (CON) across ages 3, 5, and 7. The left panel represents stable between-person components (random intercepts: BH, BD, BC), and the right panel represents within-person deviations over time. Straight arrows represent autoregressive (stability) and cross-lagged paths at the within-person level. Curved arrows represent within-wave residual covariances. Rectangular boxes represent the observed indicators at each wave, whereas the circles represent the latent within-person residual components on which the autoregressive and cross-lagged paths are estimated. Estimated within-person path coefficients are presented in Fig. 2

To model the longitudinal relations among harsh parenting, emotional dysregulation, and conduct-related externalising behaviour, analyses were conducted using a random-intercept cross-lagged panel model (RI-CLPM). This approach disaggregates stable between-child differences from within-child fluctuations over time by including random intercepts for each repeatedly measured construct (Hamaker et al., 2015). The structure of the model is illustrated in Fig. 1. Autoregressive and cross-lagged paths are estimated between the within-person residual components, which represent deviations from each child’s expected level at a given time point. Consequently, estimated associations reflect within-child change over time rather than associations driven by stable individual or family characteristics.

Within this framework, a bidirectional cross-lagged mediation model was estimated to test whether emotional dysregulation mediates associations between harsh parenting and conduct problems in both directions. The model included all autoregressive paths across waves, bidirectional cross-lagged paths between harsh parenting and emotional dysregulation and between emotional dysregulation and conduct problems at adjacent time points, and within-time residual covariances to account for contemporaneous associations not captured by lagged effects. Two indirect pathways were tested simultaneously. The parent-driven pathway examined whether harsh parenting at age 3 predicted conduct problems at age 7 indirectly via emotional dysregulation at age 5. The child-driven pathway examined whether conduct problems at age 3 predicted harsh parenting at age 7 indirectly via emotional dysregulation at age 5. By estimating both pathways within a single model, this approach allowed evaluation of whether emotional dysregulation mediates associations in one direction, both directions, or neither, and whether the magnitude of indirect effects differs across pathways.

The model was estimated using robust maximum likelihood (MLR), which provides parameter estimates and fit statistics that are robust to non-normality. Missing data were handled using full information maximum likelihood, and survey weights, stratification, and clustering variables were incorporated to account for the complex sampling design of the Millennium Cohort Study. Model fit was evaluated using standard indices (CFI, RMSEA, and SRMR). Because standard errors for indirect effects under MLR rely on the delta method—which has been shown to be conservative for mediation effects (MacKinnon et al., 1995)—indirect effects were evaluated using bootstrapped 95% confidence intervals (1,000 resamples).

To address concerns regarding measurement specification and construct validity, a sensitivity analysis was conducted in which the bidirectional mediation model was re-estimated using revised indicators, with the aim of assessing whether the key path estimates retained their sign, statistical significance, and magnitude. Specifically, the “often has temper tantrums” item was removed from the conduct problems scale because it may overlap conceptually with the emotional dysregulation item “gets easily frustrated,” potentially inflating associations due to shared affective content rather than distinct behavioural versus emotional processes. In addition, the “acts impulsively” item was removed from the emotional dysregulation scale because it may overlap conceptually with externalising behaviours, potentially inflating associations due to shared behavioural content rather than distinct emotional regulatory processes.

In addition, given that random-intercept cross-lagged panel models remain vulnerable to unmeasured time-varying confounding at the within-person level—particularly in the context of longitudinal mediation analyses—a sensitivity analysis was conducted to assess the robustness of the estimated indirect effects. Following the approach proposed by Kenny (2013) and applied in prior work using the Millennium Cohort Study (e.g., Speyer et al., 2022), “failsafe” statistics were calculated to evaluate how strong unmeasured confounding would need to be in order to attenuate the observed mediation effects to null. Specifically, the failsafe statistic estimates the magnitude of a hypothetical unmeasured confounder that would need to be equally associated with both the mediator and the outcome, conditional on the predictor, to fully explain away the indirect effect. Larger failsafe values therefore indicate that only implausibly strong unmeasured confounding could account for the observed mediation, providing greater confidence in the robustness of the findings.

### Ethical Approval

All procedures for the Millennium Cohort Study were reviewed and approved by the London Multicentre Research Ethics Committee. Written informed consent was obtained from parents at each sweep.

## Results

### Descriptive Statistics

Table 2 presents complete-case bivariate correlations among harsh parenting, emotional dysregulation, and conduct problems across ages 3, 5, and 7. Each construct showed moderate to strong stability over time, with adjacent-wave correlations ranging from *r* = .51 to *r* = .66. Cross-construct associations were also evident: harsh parenting at age 3 was positively correlated with emotional dysregulation at ages 3–7 (*r* = .21–0.29) and with conduct problems at ages 3–7 (*r* = .21–0.36). Emotional dysregulation was consistently associated with conduct problems both within and across waves, with correlations ranging from *r* = .36 to *r* = .65.

**Table 2:** Complete-case correlations among study variables

	HP3	HP5	HP7	ED3	ED5	ED7	CP3	CP5	CP7
HP3	1.00								
HP5	0.57	1.00							
HP7	0.51	0.64	1.00						
ED3	0.29	0.21	0.20	1.00					
ED5	0.23	0.31	0.28	0.55	1.00				
ED7	0.21	0.26	0.34	0.49	0.66	1.00			
CP3	0.36	0.27	0.25	0.60	0.48	0.44	1.00		
CP5	0.23	0.35	0.30	0.40	0.63	0.52	0.51	1.00	
CP7	0.21	0.28	0.37	0.36	0.51	0.65	0.45	0.61	1.00

*HP*= harsh parenting; *ED*= emotional dysregulation; *CP*= conduct problems. Numbers indicate child age (years)

### Analysis

**Fig. 2:**
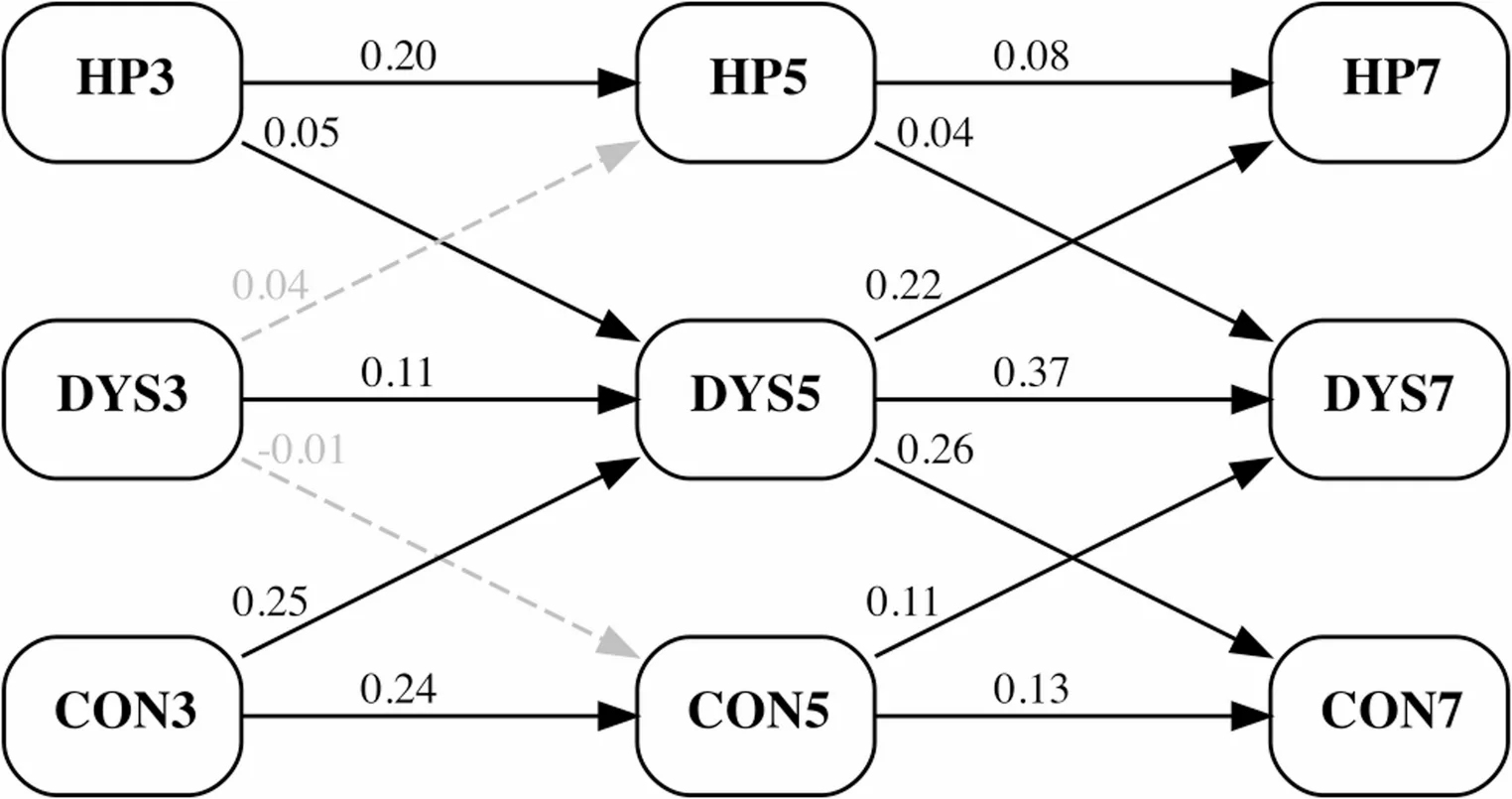
Standardised within-person path coefficients from the RI-CLPM specified in Fig. 1. Solid arrows indicate statistically significant paths (*p* < .05), and dashed grey arrows indicate non-significant cross-lagged paths. Within-wave residual covariances and between-person components (random intercepts and their covariances) are omitted for clarity. HP = harsh parenting; DYS = emotional dysregulation; CON = conduct problems. Suffixes indicate child age in years

A bidirectional RI-CLPM was estimated to examine mediation processes linking harsh parenting, emotional dysregulation, and conduct problems across ages 3, 5, and 7. Within-person cross-lagged and indirect effects are reported below; the full model structure is shown in Fig. 1. The model showed excellent fit to the data according to conventional criteria (RMSEA = 0.045, CFI = 0.99, TLI = 0.96, SRMR = 0.021). Figure 2 displays the standardized parameter estimates for all autoregressive and cross-lagged pathways in the model. (See Table S1 (Supplementary Materials 1) for regression estimates with their corresponding 95% confidence intervals for all autoregressive and cross-lagged paths.)

#### Parent-driven Mediation Pathway

Higher levels of harsh parenting were associated with subsequent increases in children’s emotional dysregulation. Harsh parenting at age 3 predicted higher emotional dysregulation at age 5 (β = 0.05, *p* < .01, 95% CI [0.02–0.08]). Emotional dysregulation, in turn, was associated with later conduct problems, with dysregulation at age 5 predicting higher conduct problems at age 7 (β = 0.27, *p* < .001, 95% CI [0.20–0.30]). The indirect effect linking harsh parenting at age 3 to conduct problems at age 7 via emotional dysregulation at age 5 was statistically significant, as indicated by bootstrapped 95% confidence intervals that excluded zero (β = 0.013, 95% CI [0.006–0.022]). This pattern indicates that associations between early harsh parenting and later conduct problems operate, in part, through children’s emotional dysregulation over time.

#### Child-driven Mediation Pathway

Evidence supported a child-driven mediation pathway. Conduct problems at age 3 predicted higher levels of emotional dysregulation at age 5 (β = 0.25, *p* < .001, 95% CI [0.215–0.286]), which in turn predicted higher levels of harsh parenting at age 7 (β = 0.22, *p* < .001, 95% CI [0.173–0.267]). The corresponding indirect effect from early conduct problems to later harsh parenting via emotional dysregulation was statistically significant, as indicated by bootstrapped 95% confidence intervals that excluded zero (β = 0.055, 95% CI [0.042–0.069]). Although this child-driven indirect effect was approximately four times larger than the parent-driven pathway (β = 0.013), both indirect effects were modest in absolute magnitude. This pattern nevertheless suggests some asymmetry in the strength of bidirectional mediation processes across early childhood. Together, these findings indicate reciprocal developmental processes in which harsh parenting and conduct problems are dynamically linked through emotional dysregulation over time.

### Sensitivity Analysis

#### Sensitivity Analysis 1: Unmeasured Time-Varying Confounding

To assess the robustness of the longitudinal mediation effects to potential unmeasured time-varying confounding at the within-child level, a failsafe sensitivity analysis was conducted. Following Kenny (2013), failsafe *ef* statistics were computed using fully standardized indirect effects. The failsafe *ef* represents the magnitude of association that an omitted time-varying confounder would need to have with both the mediator and the outcome—assuming equal effects on each path—in order to fully attenuate the estimated indirect effect to zero. Larger values therefore indicate greater robustness of the mediation effect to unmeasured confounding.

For the parent-driven pathway from harsh parenting at age 3 to conduct problems at age 7 via emotional dysregulation at age 5, the failsafe analysis indicated that an unmeasured time-varying confounder would need individual path magnitudes of approximately β = 0.50 with both the mediator and the outcome to fully attenuate the indirect effect. For the child-driven pathway, the corresponding magnitude was β = 0.46. These values are large in the context of within-person effects in RI-CLPMs, suggesting that the observed mediation effects are unlikely to be entirely driven by unmeasured confounding, though such confounding cannot be ruled out. Full calculations are provided in Supplementary Material 2, and failsafe statistics are reported in Table 3.

**Table 3:** Indirect effects from the RI-CLPM mediation model

Mediation pathway	𝛽	𝑝	95% CI	Failsafe 𝑒𝑓
HP (3) → ED (5) → CP (7)	0.013	< 0.001	[0.006, 0.022]	0.50
CP (3) → ED (5) → HP (7)	0.055	< 0.001	[0.042, 0.069]	0.46

*Notes.* Indirect effects are standardized; confidence intervals are bias-corrected (5,000 resamples)

#### Sensitivity Analysis 2: Measurement Robustness

As a further sensitivity analysis, the robustness of the mediation results was examined with respect to potential overlap in item content across constructs. Specifically, two items were identified that could plausibly blur conceptual boundaries between emotional dysregulation and conduct problems. The conduct problems item “often has temper tantrums” was removed due to its conceptual proximity to the emotional dysregulation item “gets easily frustrated”. In addition, the emotional dysregulation item “acts impulsively” was excluded, as its emphasis on overt behaviour may be more consistent with behavioural manifestations of conduct externalising problems than with internal regulatory processes.

Following these adjustments, the bidirectional mediation model was re-estimated using the modified measurement specifications. Results were highly consistent with the primary analysis. The parent-driven indirect pathway from harsh parenting at age 3 to conduct problems at age 7 via emotional dysregulation at age 5 remained statistically significant (β = 0.005), with bias-corrected bootstrapped 95% confidence intervals excluding zero (95% CI [0.002, 0.008]). Likewise, the child-driven indirect pathway from conduct problems at age 3 to harsh parenting at age 7 via emotional dysregulation at age 5 also remained robust (β = 0.043, 95% CI [0.033, 0.053]). In both cases, the direction, magnitude, and statistical significance of the indirect effects closely mirrored those obtained in the main model, indicating that the observed mediation processes are not attributable to item-level overlap or ambiguity in construct operationalisation (See Table S2 (Supplementary Materials 1) for regression estimates with their corresponding 95% confidence intervals for all autoregressive and cross-lagged paths.

## Discussion

The present study examined reciprocal longitudinal mediation processes linking harsh parenting, emotional dysregulation, and conduct problems across early to middle childhood. Using a random-intercept cross-lagged panel model applied to three waves of the Millennium Cohort Study, the analysis focused on within-child dynamics over time, isolating developmental processes from stable between-family differences. The findings provide evidence that emotional dysregulation operates as one mechanism through which both parent-driven and child-driven influences unfold across development, though the child driven pathway was larger than the parent-driven pathway.

Higher levels of harsh parenting were associated with subsequent increases in emotional dysregulation, which in turn predicted later conduct problems. Although the indirect effect was modest in magnitude (β = 0.013), it was statistically significant and robust to sensitivity analyses, indicating that part of the association between early parenting practices and later conduct problems operates through children’s difficulties regulating emotional responses over time. This pattern is consistent with developmental models proposing that harsh or punitive parenting practices may undermine children’s emerging emotion regulation capacities, thereby increasing vulnerability to behavioural difficulties (Morris et al., 2007; Sroufe & Fleeson, 2013). From an emotion socialization perspective, repeated exposure to coercive discipline may limit opportunities for children to learn adaptive strategies for managing frustration and distress within parent-child interactions, contributing to longer-term regulatory and behavioural challenges (Chang et al., 2003).

Evidence supported a child-driven mediation pathway in which earlier conduct problems were associated with subsequent increases in emotional dysregulation and, in turn, with higher levels of harsh parenting. Although the indirect effect for this pathway (β = 0.055) was larger than the corresponding parent-driven pathway (β = 0.013), both effects were relatively small in absolute terms. Even so, the difference in magnitude is consistent with transactional developmental perspectives (Sameroff, 2009), which propose that children’s behavioural difficulties can disrupt regulatory processes over time, placing strain on parent–child interactions and increasing the likelihood of more controlling or punitive parental responses. Prior work has demonstrated these links separately, with early conduct problems predicting later difficulties in emotional regulation (Eisenberg et al., 2010) and poorer emotional regulation associated with subsequent increases in harsh or controlling parenting practices (Moilanen et al., 2015). The present findings extend this work by demonstrating that emotional dysregulation mediates the pathway from early conduct problems to later harsh parenting within a longitudinal framework that accounts for stable between-family differences. This child-driven pathway contrasts with Belsky et al. (2007), who found parent-driven effects via attentional control but no evidence for reverse mediation. One explanation for this discrepancy is that emotional dysregulation—particularly in the context of early conduct problems—may evoke harsher parenting responses more strongly than deficits in cognitive regulatory capacities do. Children who exhibit frequent emotional outbursts, low frustration tolerance, and difficulty calming down may place immediate and visible demands on caregivers, increasing parental reactivity and the likelihood of coercive discipline. By contrast, cognitive regulatory skills such as attentional control may be more amenable to supportive scaffolding and less likely to trigger punitive responses, suggesting that the relative strength of bidirectional pathways may depend on which regulatory and parenting dimensions are examined.

Two complementary sensitivity analyses were conducted to evaluate the robustness of the reciprocal mediation findings. First, a failsafe analysis indicated that unmeasured time-varying confounding would need to exert moderately strong associations (*r* ≈.21–0.24) with both the mediator and the outcome to fully attenuate the observed indirect effects. In the context of within-person RI-CLPMs, confounders of this magnitude are comparable to the strongest cross-lagged effects typically observed in developmental models using large cohort studies (Speyer et al., 2022), suggesting that the observed mediation pathways are unlikely to be entirely driven by unmeasured within-child confounding, though such confounding cannot be ruled out. Second, concerns regarding potential measurement overlap were addressed by re-estimating the model under alternative measurement specifications that more sharply delineated emotional dysregulation and conduct problems. The persistence of both parent-driven and child-driven indirect pathways under these specifications—with effect sizes and significance levels closely mirroring the primary analysis—strengthens confidence that the central findings reflect substantive developmental processes rather than artifacts of construct overlap.

Although the observed indirect effects were modest in absolute magnitude — with the child-driven pathway (β = 0.055) falling between the small and medium range and the parent-driven pathway (β = 0.013) falling below the small effect threshold according to recent empirical benchmarks for cross-lagged effects (Orth et al. 2024,) — several considerations suggest that these effects may nonetheless represent meaningful developmental processes. First, effect sizes in random-intercept cross-lagged panel models are inherently constrained because they reflect within-child change over time after accounting for stable between-family differences and the considerable stability of parenting, emotion regulation, and conduct problems across early childhood (Adachi & Willoughby, 2015). In this context, even small cross-lagged associations represent deviations from expected developmental trajectories within the same child, which may accumulate and compound over successive developmental periods. Second, the magnitude of the present effects is comparable to those reported in prior longitudinal mediation studies using similar designs. For example, Sulik et al. (2015) reported indirect effects ranging from β = 0.005 to β = 0.023 in a three-wave mediation model linking parenting, executive function, and externalising behaviour, and Speyer et al. (2022) reported indirect effects as small as β = 0.002 to β = 0.004 in a random-intercept cross-lagged panel analysis of harsh parenting and child socioemotional outcomes using the same cohort. The consistency of these modest effect sizes across multiple large-scale developmental studies suggests that within-person mediation processes operating over multi-year intervals tend to produce small but replicable effects. Third, from a developmental cascade perspective, small effects at individual time points may contribute to cumulative risk processes across childhood.

Repeated cycles of harsh parenting, emotional dysregulation, and conduct problems—even if modest in magnitude at any single assessment—may become mutually reinforcing over time, with early difficulties setting in motion trajectories that become increasingly difficult to alter as children age (Masten & Cicchetti, 2010). Although the child-driven pathway observed here (β = 0.055) was larger than the parent-driven pathway (β = 0.013), both effects were modest in absolute magnitude. These findings nonetheless highlight children’s behavioural and regulatory difficulties as one potential target for early intervention.

## Limitations

Several limitations should be considered when interpreting the present findings. Although the use of a large, population-representative cohort and a random-intercept cross-lagged panel framework represents a methodological strength—particularly in its ability to disaggregate stable between-family differences from within-child change over time—important limitations remain.

First, all measures were parent-reported, with approximately 98% of reports provided by mothers. Although standard in large-scale longitudinal research, reliance on a single informant raises the possibility of shared method variance (Podsakoff et al., 2003), as mothers experiencing greater stress may simultaneously rate their own parenting, their child’s dysregulation, and their child’s behaviour more negatively. The indirect effects reported here — particularly the child-driven pathway — may therefore be partially inflated by shared reporter variance. Additionally, the findings primarily reflect maternal perceptions of parenting and child behaviour and may not generalise to father-child dynamics. Future research would benefit from multi-informant designs incorporating teacher reports, child self-reports, or observational assessments.

Second, the present study focused on conduct problems as the primary indicator of externalising behaviour. Although this subscale captures core features of oppositional and aggressive behaviuor that are theoretically most relevant to harsh parenting dynamics, the extent to which similar mediation processes operate for other externalising dimensions (e.g., hyperactivity-inattention) remains to be tested.

Third, the developmental window examined in this study spans early to middle childhood (ages 3 to 7), a period during which emotion regulation capacities are rapidly emerging and parent-child dynamics are particularly influential. It remains unclear whether similar bidirectional processes operate in adolescence, when peer relationships and autonomy-seeking may shift the balance of parent-child influence. Extending this work into later developmental stages could test whether the observed asymmetry (stronger child-driven than parent-driven effects) persists or reverses as children gain greater independence from caregivers.

Fourth, the harsh parenting measure, while widely used and psychometrically sound, captures a limited range of coercive disciplinary practices (shouting, smacking, frequent reprimands). More nuanced assessments—including observational measures or interview-based approaches—could distinguish between different forms of harsh parenting (e.g., physical vs. verbal aggression, reactive vs. instrumental coercion) and provide a richer understanding of how specific parenting behaviours relate to children’s emotional and behavioural development. As a result, the parent-driven indirect pathway identified here should be interpreted as specific to common coercive disciplinary practices rather than harsh parenting more broadly defined and may therefore not capture associations that could emerge when considering more severe or diverse forms of harsh parenting.

Finally, although the RI-CLPM reduces bias from time-invariant unobserved confounding, unmeasured time-varying factors may still influence the observed associations. Broader family stressors, contextual changes, or child-specific experiences not captured in the model could contribute to fluctuations in parenting, emotional regulation, and behaviour. Future studies incorporating richer time-varying covariates or genetically informed designs could further strengthen causal interpretations.

## Implications

Given the present findings—showing bidirectional mediation processes linking harsh parenting and conduct problems via emotional dysregulation, with somewhat larger child-driven than parent-driven effects—several implications emerge for intervention and prevention. First, the finding that child-driven effects were larger, although modest in absolute magnitude, suggests that early intervention targeting children’s conduct problems and emotion regulation difficulties may help disrupt escalating cycles of risk. Addressing early behavioural and regulatory challenges before they evoke increasingly harsh parenting responses could prevent the consolidation of coercive parent–child patterns over time. At the same time, reducing harsh or reactive parenting practices may help prevent the development of emotional dysregulation and downstream behavioural difficulties. Programs that directly build children’s emotional self-regulation skills—such as those targeting frustration tolerance, emotional reactivity, and recovery from distress—may represent one potentially useful avenue for interrupting negative cycles of parent–child interaction. Complementing these efforts, interventions that help caregivers recognize early signs of emotional dysregulation and respond in supportive, constructive ways may strengthen parent–child interactions and reduce the likelihood of harsh disciplinary responses.

Consistent with Morris et al.’s (2007) tripartite model of emotion regulation socialisation, which positions parenting practices as a primary influence on children’s developing regulatory capacities, these findings further suggest that interventions targeting both parenting behaviour and children’s emotion regulation skills during the preschool period (ages 3–6) may be particularly effective in disrupting coercive parent–child cycles. Several well-established programmes already target these dual capacities. The Incredible Years programme (Webster-Stratton, 2014) explicitly addresses harsh discipline reduction and children’s social-emotional development in group-based community and clinic settings for families with children aged 3–8. Tuning in to Kids (Havighurst et al., 2010) focuses specifically on parent emotion coaching skills, directly targeting parenting processes involved in children’s emotion regulation socialisation. The stronger child-driven pathway additionally suggests that interventions addressing children’s emotion regulation directly—such as school-based social-emotional learning curricula delivered during the transition to formal schooling—may help reduce behavioural escalation that elicits harsher parenting responses over time.

## Conclusion

This study examined longitudinal pathways linking harsh parenting, emotional dysregulation and conduct problems from ages 3 to 7. Findings showed that emotional dysregulation mediates bidirectional associations between harsh parenting and conduct problems, underscoring the transactional nature of these processes. Notably, child-driven effects (early conduct problems → dysregulation → later harsh parenting) were approximately four times larger than parent-driven effects, suggesting asymmetry in the magnitude of reciprocal pathways, although both effects were modest in absolute terms. Despite their modest size, these effects were consistent across sensitivity analyses, highlighting how small within-person influences may accumulate over time.

These results extend previous developmental cascade research by testing emotional dysregulation—rather than the cognitive self-regulation constructs more commonly examined—as a mediator linking harsh parenting and externalising behaviour, using a large, population-representative cohort and a within-person analytic framework. They also suggest that interventions may be most effective when they prioritize early efforts to address children’s conduct problems and emotion regulation difficulties—given the magnitude of child-driven effects—while also supporting positive parenting practices to disrupt escalating cycles of risk.

## Supplementary Information

Below is the link to the electronic supplementary material.

Supplementary Material 1 (PDF 197 KB)

Supplementary Material 2 (DOCX 21.9 KB)

## Data Availability

The data used in this study are drawn from the Millennium Cohort Study, which is administered by the University of London Centre for Longitudinal Studies and archived with the UK Data Service. Access to these data is subject to registration and approval through the UK Data Service, in accordance with their data access procedures (https://ukdataservice.ac.uk).
