# Supplementary material for: Reciprocal Pathways Linking Harsh Parenting and Conduct Problems in Early Childhood: The Mediating Role of Emotional Dysregulation
Source: Res Child Adolesc Psychopathol. 2026 Jul 4;54(4):87. doi: 10.1007/s10802-026-01473-8 (PMC13332879; doi:10.1007/s10802-026-01473-8)
Supplement: Supplementary file 1 — Supplementary Material 1 (PDF 197 KB) [file 10802_2026_1473_MOESM1_ESM.pdf]

Supplementary Information 2: Failsafe Sensitivity Analysis

Ricardo Mellado Labbé

2026-02-04

Supplementary Materials 2

1 Sensitivity Analysis for Unmeasured Time-Varying Confounding

To evaluate the robustness of the estimated longitudinal mediation effects to unmeasured time-varying confounding at the within-child level, a sensitivity analysis based on the failsafe ef statistic was conducted (Kenny, 2013). This approach quantifies the magnitude of association that an omitted time-varying factor would need to have simultaneously with the mediator and the outcome—conditional on the predictor—in order to fully attenuate the estimated indirect effect.

The logic underlying the failsafe ef statistic is that any unmeasured confounder capable of explaining away a mediation effect must exert non-trivial effects on both the mediator (e) and the outcome (f). The product ef therefore represents the joint strength of these associations required to reduce the indirect pathway to zero.

The standardized failsafe ef was computed using:

$$\text{failsafe } ef = \frac{r_{MY\cdot X}\, S_{M\cdot X}\, S_{Y\cdot X}}{S_M\, S_Y}$$

where $r_{MY\cdot X}$ denotes the standardized regression coefficient for the association between the mediator (M) and the outcome (Y), conditional on the predictor (X). In the present models, this coefficient additionally reflects adjustment for all other variables with direct paths to the outcome. $S_{M\cdot X}$ and $S_{Y\cdot X}$ represent the residual standard deviations of the mediator and outcome after accounting for the predictor, and S_M and S_Y are the total standard deviations of the mediator and outcome, respectively.

For interpretability, variables were standardized such that $S_M = S_Y = 1$. Under this parameterization, the residual standard deviations were obtained as:

$$S_{M\cdot X} = \sqrt{1 - r_{MX}^2} \quad \text{and} \quad S_{Y\cdot X} = \sqrt{1 - r_{MY\cdot X}^2}$$

where r_{MX} is the standardized coefficient for the association between the predictor and the mediator.

Following Kenny (2013), the magnitude of the hypothetical confounding paths e and f was derived by assuming equal strength of association with the mediator and outcome ($e = f$), such that:

$$e = f = \sqrt{\text{failsafe } ef}$$

Larger values of e (and f) indicate that only very strong unmeasured time-varying influences could fully account for the observed indirect effect.

2 Failsafe calculation

The following calculations illustrate the application of the failsafe ef statistic to the two mediation pathways estimated in the present study. All input values are fully standardized regression coefficients (STDYX) extracted from the Mplus RI-CLPM solution.

2.1 Parent-driven pathway: HP(age 3) → ED(age 5) → CP(age 7)

The standardized path from harsh parenting at age 3 to emotional dysregulation at age 5 was $r_{MX} = 0.051$, and the standardized path from emotional dysregulation at age 5 to conduct problems at age 7, conditional on all other predictors in the model, was $r_{MY \cdot X} = 0.256$. Under standardization ($S_M = S_Y = 1$), the residual standard deviations were:

$$S_{M \cdot X} = \sqrt{1 - 0.051^2} = 0.9987$$

$$S_{Y \cdot X} = \sqrt{1 - 0.256^2} = 0.9667$$

The failsafe statistic was therefore:

$$\text{failsafe } ef = \frac{0.256 \times 0.9987 \times 0.9667}{1 \times 1} = 0.247$$

Assuming equal confounding paths ($e = f$):

$$e = f = \sqrt{0.247} = 0.497$$

2.2 Child-driven pathway: CP(age 3) → ED(age 5) → HP(age 7)

The standardized path from conduct problems at age 3 to emotional dysregulation at age 5 was $r_{MX} = 0.251$, and the standardized path from emotional dysregulation at age 5 to harsh parenting at age 7, conditional on all other predictors, was $r_{MY \cdot X} = 0.220$.

The residual standard deviations were:

$$S_{M \cdot X} = \sqrt{1 - 0.251^2} = 0.9680$$

$$S_{Y \cdot X} = \sqrt{1 - 0.220^2} = 0.9755$$

The failsafe statistic was therefore:

$$\text{failsafe } ef = \frac{0.220 \times 0.9680 \times 0.9755}{1 \times 1} = 0.208$$

Assuming equal confounding paths ($e = f$):

$$e = f = \sqrt{0.208} = 0.456$$

References

Kenny, D. A. (2013). *Mediation: Sensitivity analysis.* http://davidakenny.net/webinars/Mediation/Sensitivity/Sensitivity.html
