# Supplementary material for: Reciprocal Pathways Linking Harsh Parenting and Conduct Problems in Early Childhood: The Mediating Role of Emotional Dysregulation
Source: Res Child Adolesc Psychopathol. 2026 Jul 4;54(4):87. doi: 10.1007/s10802-026-01473-8 (PMC13332879; doi:10.1007/s10802-026-01473-8)
Supplement: Supplementary file 2 — Supplementary Material 2 (DOCX 21.9 KB) [file 10802_2026_1473_MOESM2_ESM.docx]

Supplementary Information

# Supplementary table 1

Table 1: Regression coefficients for all paths in the RI-CLPM (original measurement specification)

| **Path** | 𝛽 | **SE** | **95% CI** |
| --- | --- | --- | --- |
| **Autoregressive paths**  HP3 → HP5 | 0.20*** | 0.02 | [0.17, 0.24] |
| HP5 → HP7 | 0.08* | 0.04 | [0.01, 0.16] |
| DYS3 → DYS5 | 0.11*** | 0.03 | [0.05, 0.17] |
| DYS5 → DYS7 | 0.37*** | 0.02 | [0.33, 0.41] |
| CON3 → CON5 | 0.24*** | 0.02 | [0.20, 0.29] |
| CON5 → CON7 | 0.13*** | 0.04 | [0.05, 0.20] |
| **Cross-lagged paths**  HP3 → DYS5 | 0.05*** | 0.02 | [0.02, 0.08] |
| HP5 → DYS7 | 0.04** | 0.02 | [0.01, 0.08] |
| DYS3 → HP5 | 0.04 | 0.02 | [-0.01, 0.10] |
| DYS5 → HP7 | 0.22*** | 0.03 | [0.17, 0.27] |
| CON3 → DYS5 | 0.25*** | 0.02 | [0.21, 0.29] |
| CON5 → DYS7 | 0.11*** | 0.02 | [0.07, 0.15] |
| DYS5 → CON7 | 0.26*** | 0.03 | [0.21, 0.31] |

*Note*. HP = Harsh Parenting; DYS = Emotional Dysregulation; CON = Conduct Problems. Coefficients are standardized within-person estimates. CI = confidence interval. ^∗^𝑝 < .05, ^∗∗^𝑝 < .01, ^∗∗∗^𝑝 < .001.

# Supplementary table 2

Table 2: Regression coefficients for all paths in the RI-CLPM under alternative measurement specifications used in the sensitivity analysis, with 95% confidence intervals

| **Path** | 𝛽 | **SE** | **95% CI** |
| --- | --- | --- | --- |
| **Autoregressive paths**  HP3 → HP5 | 0.20*** | 0.02 | [0.16, 0.24] |
| HP5 → HP7 | 0.08* | 0.04 | [0.01, 0.15] |
| DYS3 → DYS5 | 0.12*** | 0.03 | [0.07, 0.17] |
| DYS5 → DYS7 | 0.34*** | 0.02 | [0.31, 0.39] |
| CON3 → CON5 | 0.23*** | 0.02 | [0.11, 0.17] |
| CON5 → CON7 | 0.11** | 0.04 | [0.04, 0.20] |
| **Cross-lagged paths**  HP3 → DYS5 | 0.05** | 0.02 | [0.00, 0.02] |
| HP5 → DYS7 | 0.06** | 0.02 | [0.01, 0.03] |
| DYS5 → HP7 | 0.21*** | 0.02 | [0.52, 0.81] |
| CON3 → DYS5 | 0.24*** | 0.02 | [0.04, 0.06] |
| CON5 → DYS7 | 0.10*** | 0.02 | [0.02, 0.05] |
| DYS5 → CON7 | 0.24*** | 0.03 | [0.56, 0.82] |

*Note*. HP = Harsh Parenting; DYS = Emotional Dysregulation; CON = Conduct Problems. Coefficients are standardized within-person estimates. CI = confidence interval. ^∗^𝑝 < .05, ^∗∗^𝑝 < .01, ^∗∗∗^𝑝 < .001.
